# Rumination as a Transdiagnostic Phenomenon in the 21st Century: The Flow Model of Rumination

**DOI:** 10.3390/brainsci13071041

**Published:** 2023-07-08

**Authors:** Stephanie M. Y. Wong, Eric Y. H. Chen, Michelle C. Y. Lee, Y. N. Suen, Christy L. M. Hui

**Affiliations:** 1 Department of Psychiatry, School of Clinical Medicine, LKS Faculty of Medicine, The University of Hong Kong, Hong Kong; steph.my.wong@gmail.com (S.M.Y.W.); leecymichelle115@gmail.com (M.C.Y.L.); suenyn@hku.hk (Y.N.S.); christy@lmhui.com (C.L.M.H.); 2 The State Key Laboratory of Brain and Cognitive Sciences, The University of Hong Kong, Hong Kong

**Keywords:** rumination, flow model of rumination, cognition, technological advances

## Abstract

Rumination and its related mental phenomena share associated impairments in cognition, such as executive functions and attentional processes across different clinical conditions (e.g., in psychotic disorders). In recent decades, however, the notion of rumination has been increasingly narrowed to the “self-focused” type in depressive disorders. A closer review of the literature shows that rumination may be construed as a broader process characterized by repetitive thoughts about certain mental contents that interfere with one’s daily activities, not only limited to those related to “self”. A further examination of the construct of rumination beyond the narrowly focused depressive rumination would help expand intervention opportunities for mental disorders in today’s context. We first review the development of the clinical construct of rumination with regard to its historical roots and its roles in psychopathology. This builds the foundation for the introduction of the “Flow Model of Rumination (FMR)”, which conceptualizes rumination as a disruption of a smooth flow of mental contents in conscious experience that depends on the coordinated interactions between intention, memory, affect, and external events. The conceptual review concludes with a discussion of the impact of rapid technological advances (such as smartphones) on rumination. Particularly in contemporary societies today, a broader consideration of rumination not only from a cognition viewpoint, but also incorporating a human–device interaction perspective, is necessitated. The implications of the FMR in contemporary mental health practice are discussed.

## 1. Introduction

The phenomenon of rumination has long been recognized as a symptom of psychiatric conditions. Rumination may generally be construed as a process of *repetitive thoughts* [1]. While rumination was described as “fixed ideas” (French: *idees fixes*) and the “compulsion to repeat” (German: *Wiederholungszwang*) [2] in the 19^th^ century, recent research has increasingly confined the phenomenon to a narrowed focus on rumination of the *depressive* type [3,4,5]. As a result, the study of the mechanisms underlying rumination may have been limited to a narrower construct of depressive rumination. Such a focus might have inadvertently limited a broader understanding of its phenomenology and its application to mental health work and research. 

Narrowed attention span and reduced executive capacity have been suggested to play critical roles in rumination and a broader range of mental health conditions [6,7]. In addition, in contrast to depressive rumination, the content of rumination can consist of not only the self but also environmental factors [8,9,10,11]. It is recognized that rumination is a transdiagnostic symptom observed across psychiatric conditions, including not only depression and generalized anxiety but also post-traumatic stress disorder (PTSD), social anxiety disorder, obsessive compulsive disorder, bipolar disorder, and psychosis spectrum disorders [12,13,14,15]. 

A broader investigation into the mechanisms underlying rumination not limited to the depressive type can help widen future intervention opportunities for some of the most complex and debilitating psychiatric conditions. We begin with a conceptual review of existing accounts of rumination and its related phenomena in this paper, followed by the introduction of a comprehensive integrative framework—the Flow Model of Rumination (FMR)—wherein rumination is conceptualized as a neurocognitive process that encompasses a wide range of experiences related to the “*flow*” of mental contents. From this perspective, rumination may be understood not only as a form of internally triggered self-focused attention but also as *“outward-focused”* attention. This renewed reflection on rumination is built upon both earlier and recent work to offer a parsimonious account of both internally and externally focused ruminative experiences. Importantly, the increasing availability of mobile online technologies [16,17,18,19] has provided additional mechanisms for triggering and maintaining ruminative experiences, which has important implications for modern challenges in global mental health. The potential application of the FMR in contemporary mental health practices in today’s context will also be discussed. 

## 2. Existing Accounts of Rumination

### 2.1. Early Accounts of Rumination 

The notion that ideas flow in succession by “connexion and association” [20] has been implicit in major accounts of human thought processes from the writings of Aristotle—see Buckingham and Finger (1997) [21]—as well as Hobbes (1651) [22], Locke (1689) [23], and Hume (1748) [24]. These perspectives laid the foundations for the account of the “stream of consciousness” in James (1890) [25], which characterized different modes of “forward-flowing ideas”. On this basis, more recent theories have defined “mental progression” as the smooth advancement of relevant mental contents *embedded within coherent contexts* (e.g., [26]), which presumes spontaneous progression unless there is a deliberate intent to “focus” on the idea [24,25,26,27,28]. By the 1800s and 1900s, the excessive and involuntary tendency to fixate on certain thoughts and ideas had also been described as *“idee irresistible”* [29] and *“idees fixes”* [30]. These early formulations of repetitive internal experiences shed light on the relevance of intentionality and regulatory processes in the genesis of experiential contents and draw similarities with recent neuroscientific research on the role of mental representations in thought suppression [31].

### 2.2. Recent Accounts of Rumination 

#### 2.2.1. Models of Depressive Rumination 

Various models of depressive rumination have since been proposed. The Response Styles Theory (RST) [3] describes rumination as repetitive thoughts about one’s distress symptoms and their causes and consequences. This type of *self-focused* rumination is also termed “brooding”, which is characterized by thoughts such as “*Why did this happen to me and not others?*”, *“What am I doing to deserve this?”*, and *“Why do I have problems other people don’t have?”* [32]. Similarly, the Rumination on Sadness model that was later postulated [33] also conceptualized ruminative thoughts as being fixated on *negative affect* rather than being goal-directed. The H-EX-A-GO-N model of rumination [34], which considers the individual components of Habit development, Executive control, Abstract processing, Goal discrepancies, and Negative bias, is among the latest efforts to organize existing models with the aim to better understand the onset and maintenance of depressive rumination, although its focus is also limited to the self-focused type of rumination. Rumination about *non*-self-focused contents, but rather those concerning other people and environmental stressors, has been less discussed. 

#### 2.2.2. Goal Progress Theory of Rumination 

Several other models have considered the relevance of rumination to other personal intentions and internal mechanisms, such as one’s goals, over-general memory, and spontaneous thoughts. *Goal-oriented rumination* is another example of a commonly studied form of rumination that has been described in the Goal Progress Theory of Rumination. This type of rumination contrasts with depressive rumination in that its thought content is focused not on depressive symptoms but rather on one’s failure to make progress towards personally significant goals [35]. In addition, while negative affect can also arise as a result of goal failure, the maintenance of goals in one’s awareness may also facilitate problem-solving, self-evaluation, and, in turn, goal attainment. Goal-oriented rumination may thus at times also be considered to be a healthy adaptive form of rumination. Nevertheless, there is still little understanding of *when* goal-oriented rumination can be beneficial or detrimental to mental health. This theory also puts more emphasis on mental contents that are directly related to oneself, with little consideration for how one’s rumination state can be affected by one’s external environment. 

#### 2.2.3. Accounts of Rumination in Relation to External Events

The Stress-Reactive Model of Rumination [36] and the Post-Event Processing (PEP) theory [37] are two examples of rumination that also consider the relevance of *external events* in one’s rumination content. *Stress-reactive* rumination refers to repetitive thoughts about the negative inferences arising from stressful experiences [36]. Interestingly, longitudinal and ecological momentary assessment studies have found that stress-reactive rumination, but not self-focused depressive rumination, can interact with external life stressors to increase depressive symptoms [38,39]. Nonetheless, as exemplified by its measurement instrument (the Stress-Reactive Rumination Scale [39]), this form of rumination remains largely “self-focused” in that its content largely revolves around oneself rather than the event (e.g., “*Think about how the stressful event was all your fault*,” “*Think about how things like this always happen to you*”). 

Meanwhile, the PEP theory postulates that those with social anxiety tend to internally review their behaviors following engagement in distressing social events [37,40]. The excessive encoding of negative details related to one’s performance can result in negative self-perceptions after the event. Thoughts related to whether one “could have done differently” in the situation (self-focused) also appear to play a bigger role in PEP as compared to details of the event. 

#### 2.2.4. Neurocognitive Models of Rumination 

Various neurocognitive models have also been proposed with the aim of explaining rumination and its underlying cognitive mechanisms. Their explanations of the phenomenon and its roles provide a relatively broader framework that can also be applied to other psychiatric conditions. 

For instance, the “Capture and Rumination, Functional Avoidance, and Executive Control” (CaRFAX) Model hypothesized that the activation of important, though unpleasant, conceptual self-relevant information could “capture” one’s attention and increase the retrieval of related autobiographical memories [41]. This may also be understood in the context of psychotic symptoms, such as “ideas of reference” (i.e., attribution of often irrelevant information in the social environment as targeting oneself), which have often been discussed in relation to attentional biases to threatening stimuli [42,43,44,45]. Attentional biases to and “attentional capture by” threat, including emotionally salient threat signals (e.g., angry faces), have also been recorded in those with prior trauma exposure and PTSD [46,47,48]. According to the CaRFAX model, the fixation on selected thoughts and the further diminishing of executive resources could also contribute to overgeneral memories [49,50], and it has been shown to be implicated in not only depression but also PTSD and psychosis spectrum disorders [51,52,53,54]. 

The Attentional Scope Model of Rumination views rumination as a narrowed array of thoughts, percepts, and actions in one’s working memory or long-term memory. This in turn leads to the continuation of focus on certain thought contents and difficulties in shifting [7]. From this perspective, rumination may be viewed as a more general form of perseverative cognition. 

Indeed, several researchers have viewed rumination as a process characterized by strong automatic constraints and limited mental flexibility relative to daydreaming and mind-wandering [27,55,56,57]. Recent neurobiological accounts have also identified altered connectivity of the default mode network (DMN) to be associated with rumination [58,59], which has also been considered to underlie the self-referential processes seen in other mood and anxiety disorders, PTSD, and psychotic disorders [60,61,62,63]. 

Importantly, in addition to the focus on rumination as a marker of increased cognitive vulnerability to depressive disorders, an abundance of studies have in fact provided evidence to suggest its impact on anxiety disorders [64,65,66], as well as other psychiatric conditions including PTSD, social anxiety disorder, OCD, bipolar disorder, and psychotic disorders [13,67,68,69,70]. These studies provide a strong basis to consider rumination beyond the narrowly defined depressive type. 

#### 2.2.5. Neurobiological Accounts of Rumination and Related Processes

One example of ruminative processes is the phenomenon of “obsessions” in OCD, which are described as self-generated intrusive repetitive thinking that is difficult to control and disengage from [71,72]. Increased activity has been detected in functional imaging studies in the orbitofrontal cortex, the anterior and posterior cingulate gyrus, the insula, the caudate nucleus, and the anterior thalamus regions [73]. These components, particularly involving the cortico-striato-thalamo-cortical loop, are linked to obsessive thoughts (e.g., [74,75,76]) in OCD, the increase in which may be normalized with treatment (e.g., selective serotonin reuptake inhibitors). Another potentially relevant brain process in rumination may involve perseveration, which refers to the persistence of a previously appropriate response that is no longer appropriate due to the change in context. The inability to shift-set and switch responses relevant to the context may be related to the failure of multiple inhibitory systems in the lateral prefrontal cortex [77]. It is thus likely that ruminative processes are related to multiple excitatory and inhibitory brain mechanisms in the prefrontal cortex as well as the cortico-striato-pallidal-thalamic loops. 

### 2.3. Rumination beyond Depressive Disorders 

#### 2.3.1. Post-Traumatic Stress Disorder 

In fact, one of the earlier works that formed the foundations of RST examined the ruminative response styles in both depressive and PTSD symptoms following the 1989 Loma Prieta Earthquake [8]. Measures of the study consisted of not only depressive rumination but also rumination *about the earthquake* (e.g., thoughts about “*the moment the earthquake happened*,” “*the people who were killed*,” and “*what might have happened during the earthquake*”)—a type of “event-based rumination”. Beyond depressive rumination, rumination in PTSD has been defined as repeated thoughts about a traumatic event, its consequences, and the retrospective possibilities of prevention [69,78]. More recent studies have also confirmed the manifestation of this type of event-based rumination subsequent to significant stressors and trauma and have confirmed its associations with not only symptoms of depression but also PTSD and ideas of reference [9,79,80,81,82]. 

Notably, the study on survivors of the 2008 Wenchuan earthquake found that intrusive rumination about the earthquake (e.g., “*I cannot help thinking about the earthquake*”) was associated with PTSD symptoms, whereas *deliberate* rumination about the earthquake (e.g., “*I thought about what I could learn from the earthquake experience*”) was associated with symptoms of post-traumatic growth (e.g., realization and appreciation of personal strength and life and new possibilities) [82], which further suggest differential pathways between involuntary and voluntary ruminative thoughts, with the latter showing more resemblance to constructive repetitive thinking and goal-oriented rumination. 

#### 2.3.2. Rumination in Suicidal Ideation and Related Behaviors 

Of note, while depressive rumination has also been found to be a precursor of suicidal ideation and behaviors [83,84], a number of studies have discussed the phenomenon of perseverative cognitions *about suicide* (termed “suicide-specific cognitions” or “suicide-related rumination”), which describes persistent thoughts about suicide to the extent that current tasks are affected [85,86,87,88,89]. Suicide-related rumination offers an example of fixed thoughts about an intention. Although suicidal thoughts are closely related to depression, further studies of this important phenomenon may benefit from a broadened framework from the narrowly focused depressive rumination. 

#### 2.3.3. Psychotic Symptoms and Disorders

Perseverative thinking and difficulties in set-shifting, representing other related forms of perseverative cognitions, have also long been linked to prefrontal dysfunctions in schizophrenia [90,91]. Like rumination, perseveration involves unintentional yet persistent and context-inappropriate thoughts [92], although multiple modalities can be implicated, spanning beyond semantic repetitions to perseverative cognition and motor phenomena [93]. Of note, using a “content-free” measure of perseverative thoughts (e.g., “*I keep thinking about the same issues all the time*” in the Perseverative Thinking Questionnaire [94]), a study has reported significantly higher levels of negative perseverative thoughts in patients with persecutory delusions as compared to healthy controls [95]. At the same time, preoccupation has been considered to be a key dimension of delusions and related experiences (e.g., [96,97,98,99]) and is also directly relevant to experiences of paranoia and hallucinations in subthreshold psychotic-like experiences [100]. 

Indeed, rumination has been linked to proneness to psychosis and psychosis disorders, plausibly via the fixation on negative information and excessive cognitive load [101,102,103]. A study conducted among remitted schizophrenia patients has in fact found that rumination was associated with negative symptoms—particularly stereotyped thinking and emotional withdrawal—but *not* depressive symptoms [104]. This suggests that rumination may be differentially manifested across psychiatric conditions. In another study that explored the *content* of rumination in a group of schizophrenia patients, 70% of patients perceived their ruminative thoughts to be related to their mental illness, with some noting contents of not only “life failure” and significant life events but also their psychotic symptoms, such as “voices” [105]. A recent study conducted amid large-scale social unrest and COVID-19 in Hong Kong has also reported event-based rumination to be significantly associated with the psychotic-like experience of ideas of reference in a large community sample [81].

## 3. The Flow Model of Rumination

Based on the aforementioned gaps in existing models of rumination, we propose the Flow Model of Rumination (FMR) to broaden the understanding and applicability of the phenomenon. The notion of “flow” in the FMR differs from that described by Csikszentmihalyi (1975) (i.e., an “optimal” state of consciousness characterized by focused concentration of one’s present experience) [106] but rather places emphasis on the “flow of thoughts” with time (e.g., the moment-to-moment awareness and thought contents), likened to that described in the work of Klinger and Cox (1987) and other neurocognitive models of mental states and experiences (e.g., [57,107,108]).

**Figure 1:**
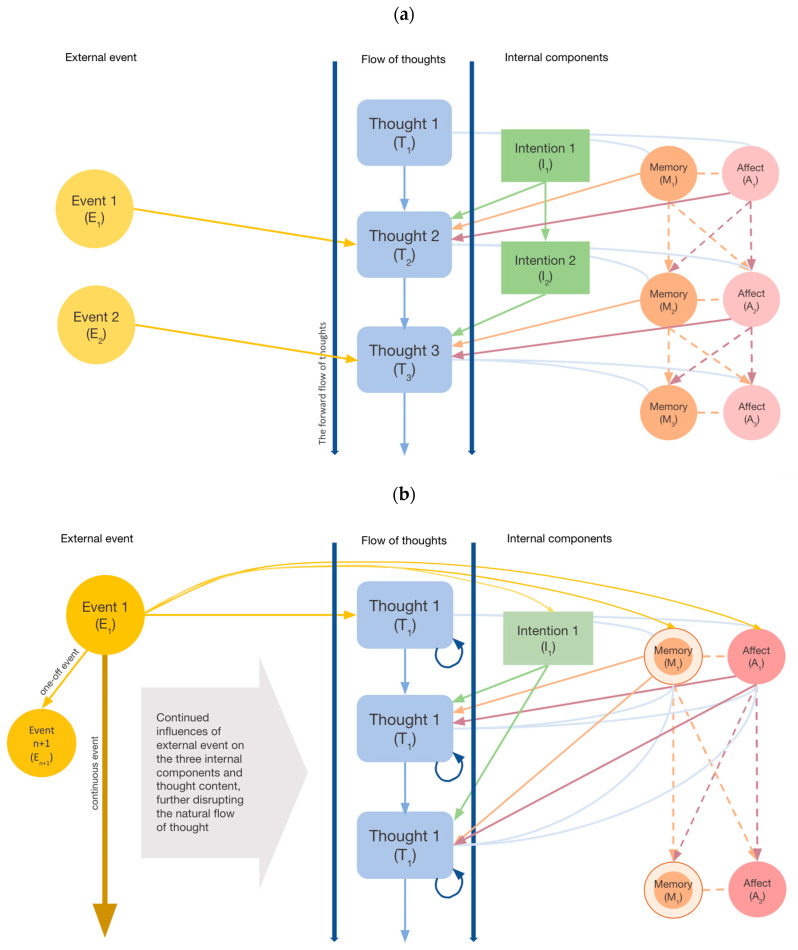
The Flow Model of Rumination under normal and stressful circumstances. (**a**) Ruminative experiences under normal circumstances. (**b**) Ruminative experiences under stressful situations.

In the FMR, a smooth flow of content of consciousness results from coordinated interactions between three internal components and one external component. Experiential content at a future time point is determined by contents at *T*_*N*+1_, namely intention (I), memory (M), affect (A), and external events (E) at the previous time point (Figure 1). Figure 1a illustrates the forward-regulated flow of thoughts in the absence of rumination, while Figure 1b illustrates the stagnated flow of thoughts (state of rumination) in the presence of significant external events. These components are expected to interact in a dynamic manner and involve mechanisms including intentional activation, temporal decay, attentional shifts, affective reactivity, and inhibitory processes in regulating the “flow” of thoughts. It is also postulated that each experiential content at the *present* moment contributes as a source of input that partially determines the mental content at *the next moment* (*T*_*N*+1_). Details of each of these components are further described below. 

This model stands out from existing concepts of mental progression in highlighting the combined influences of both internal cognitive processes, affective states, and the external environment in determining whether a thought would “flow” or become stagnated (presented as two extremes on a continuum), as in the case of rumination.

*Note.* In Figure 1a, the Flow Model of Rumination (FMR) illustrates four key factors contributing to the forward-regulated flow of thoughts in the absence of rumination. On the left are conscious processes, including the influence of external events and deliberate intentions over subsequent thoughts (*T*_*N*+1_). The right side depicts the relationship between moving thoughts and affective states and preconscious memory. Figure 1b illustrates the occurrence of rumination in the presence of a significant external event. The event E_1_, which may either be a one-off incident or an ongoing situation, can feed into persistent event-related thought in spite of other events occurring in the environment (*E*_*n*+1_). Such an event can also stimulate certain affective states, activate certain preconscious memory deemed to be associated with the current experiences (which could be spurious in some conditions), and influence the intent to repeatedly focus on the same thought. All these processes also depend on one’s cognitive state and attentional control. Together, external events and internal processes can stagnate thought progression, which can result in a process of rumination. 

### 3.1. Intentional and Involuntary Activation

One key component of the model concerns the intentional or involuntary modes of thought maintenance (Figure 1). The intention to *actively* focus on specific thoughts is typically involved in task-relevant, goal-directed problem-solving [35,109,110]. While typical problem-solving situations require a degree of cognitive flexibility to accommodate alternative thinking [111], *involuntarily* triggered thought contents can increase the likelihood of fixated thoughts. Indeed, *incidental*, as compared to intentional, encoding and consolidation of information have been found to be associated with poorer attentional control [112]. Neuroimaging observations have also suggested that intentional thinking is associated with Task Positive Network activations as opposed to DMN activities [113]. 

### 3.2. Affect and Interactions with Cognitive Processes and Memory 

The experience of different affective states can be associated with stagnation in thoughts in different manners and may relate to the form of attentional biases present across psychiatric conditions. 

#### 3.2.1. Negative Affect

The role of negative affect in exacerbating ruminative thoughts has been elaborately discussed in the literature. Those with more depressive symptoms have been shown to have a greater tendency to focus their attention on negatively valenced information [32,34,114], which can, in turn, contribute to increased preoccupations and narrowed attention [3,7,115,116,117,118]. 

#### 3.2.2. Angry Affect

Aside from negative affect, angry affect (e.g., after a traumatic event, being provoked) has also been found to be a key factor that contributes to *rumination* [10,119]. In a brain imaging study, significant activation in areas such as the hippocampus, insula, and cingulate cortex has been found to be associated with angry rumination following provocation [10]. Despite significant correlations between anger and depressive rumination [120], several studies have found *anger* rumination to be at least partially distinct from *depressive* rumination, wherein anger rumination is uniquely associated with aggression and externalizing disorders in females [121,122].

#### 3.2.3. Positive Affect

While less frequent, it is also possible that individuals also dwell on positive aspects of themselves and positive life events [123,124]. This type of “positive rumination” has received less scientific attention, although a number of studies have found that engagement in positive rumination is in fact associated with fewer depressive symptoms [125] and improved working memory-updating ability [126,127]. 

It is indeed possible that “positive rumination” requires effort to fixate on positive thoughts (as in the case of constructive repetitive thinking [128]). Notably, some aspects of positive rumination (e.g., emotion-focused rather than self-focused) may be more pronounced in conditions such as mania [11]. This may also relate to an increased attentional bias to positive stimuli, avoidance of negative stimuli, and difficulties in focusing attention [129,130,131,132]. 

#### 3.2.4. Suspiciousness and Paranoia 

Furthermore, particularly in psychotic disorders, increased experiences of suspiciousness can be triggered by external stressors [133,134]. These experiences can interact with altered cognitive processes, such as heightened attention and prolonged fixation on threatening cues and negative information [114,135,136,137], reduced cognitive flexibility [138], working memory deficits [139], over-general autobiographical memory [140], reduced memory specificity and richness of detail [141], and other negative affective states (e.g., social anxiety, generalized anxiety, depressive mood) [142]. Notably, the tendency to attend to perceived threatening stimuli and make spurious associations between coincidental thoughts and environmental cues [143] may further perpetuate the cycle of fixated thoughts related to delusional ideas. 

### 3.3. Interactions between Internal and External Influences in the Flow of Mental States 

In the FMR, mental contents activated during a rumination-free state tend to fade with time as new content emerges [144,145,146]. The constant dynamic interplay between intrinsic and extrinsic factors determines the “flow” of mental states. 

In situations where the external environment is relatively stable, the contribution to the stagnation in thoughts would largely be the result of dysregulations of internal cognitive processes (e.g., reduced attentional resources and cognitive biases [1]). For instance, the failure of inhibitory processes can compromise disengagement from repetitive thoughts in the working memory and limit the emergence of new mental contents [147,148]. At the same time, the failure to update mental representations is also considered to be one of the key processes underlying a stagnated mental flow state [149] and deficits in inhibiting mental representations of the previous moment [150]. Notably, as affect often does not fade quickly and is susceptible to reactivation and intensification [119,151,152], the persistence of affect-laden thoughts can further interfere with information updating and increase the likelihood of rumination [153]. 

However, when faced with significant, intense triggers in the environment (e.g., traumatic, threatening events, and large-scale stressors), particularly those that do not integrate well with one’s pre-existing worldviews [154,155], the flow of thoughts could become disrupted even when internal processes are relatively intact. 

Exposure to significant stressors could activate pre-existing memories and intensify the affective experiences [151], which can in turn perpetuate the rumination cycle (Figure 1b). The activation of mental representations of significant events is also often emotionally intense and value-laden [156], which can make disengagement challenging. The experience of “event-based rumination” has been discussed in the realms of personal events [10,157], socio-political instabilities [9,158], and the collective experiences of natural disasters and pandemics [3,159].

## 4. The Experience of Rumination in the 21st Century

The increasing reliance on digital technologies today may offer a new avenue that further promotes rumination. The abundant flow of largely unfiltered and externally organized online information from the Internet into individual awareness can not only increase one’s cognitive load but also increase the possibility of receiving highly triggering salient information [19,160,161,162,163,164], both of which can contribute to the activation and maintenance of ruminative thoughts.

While smartphone use may first appear to be a coping strategy in distracting one from negative real-life experiences [165], studies have shown that increased *dependence* on the Internet and smartphones is in fact associated with alterations in neurobiological structures and cognitive deficits, as well as poorer mental health [161,164,166]. The use of digital devices for “escaping” from negative affective states could also increase the risks for attentional biases (e.g., searching for more negative contents, bias toward negatively valenced content online), which may further promote ruminative thoughts. Indeed, exposure to some online information, such as crime scenes, accidents, and conflicts and public health crises such as COVID-19, have been reported to increase not only depressive mood but also anger and fear [167,168,169]. The link between Internet and smartphone use and repetitive thoughts has been reported [170,171,172]. The continuous looping and passive intake of online information on smartphones in fact also show resemblance to the difficulties in breaking away from locked cycles of thoughts and behaviors in rumination. The fear of online surveillance and other related self-referential and delusional thoughts have also been reported in several studies [117,173,174], which can possibly trigger rumination in those with higher paranoid affect.

From the perspective of the FMR, the reliance on smartphone technologies today acts as an external mechanism that not only prolongs the exposure and effects of one-off external stressors but also reduces one’s intentionality in one’s behaviors, reinforces one’s affective states and memory of the past event, and depletes one’s cognitive resources, thereby altogether increasing the stagnation in the flow of thoughts.

As discussed, the narrowing of research focus towards depressive rumination took off in the 1990s before the advent of smartphones, which have critically transformed human interactions with social and digital environments [19,175]. Re-conceptualizing rumination in the age of digitalization by taking into account the extensive role of digital information in everyday experiences is required. 

## 5. Opportunities for Intervention: Current Work and Future Directions

Various interventions have been designed for depressive rumination. For instance, rumination-focused cognitive behavioral therapy (RFCBT) has been noted to be effective in improving depressive rumination as well as mood and anxiety disorders [176,177,178]. Elements of behavioral activation, including the identification of how and when rumination occurs, the potential triggers of rumination, its possible function, behavioral experiments (e.g., limiting future exposure and practicing alternative habits in response to such cues), and the devising of specific plans to remove or replace the ruminative thoughts, have been identified to facilitate intervention outcomes [34]. In addition, mindfulness-based cognitive therapies (MBCTs) have also been used to reduce rigid repetitive thinking [179,180], aiming to disengage and redirect individuals’ attention from automatic ruminative content to their stream of conscious bodily experiences [181,182]. It has also been noted that supplementing attentional training with CBT can further enhance cognitive flexibility [183], which may be useful for reducing rumination. 

With relevance to the FMR, interventions may be designed to interrupt the stagnation in thoughts with consideration of each of the following four components: intention (I), memory (M), affect (A), and external events (E). Elements integral to RFCBT and MBCT may be utilized, given that such approaches can help redirect one’s attention from originally fixated thoughts. Other studies have shown that cognitive-based treatments targeting attentional bias, reasoning, and interpretation, as well as cognitive remediation, are effective in reducing rumination [184] *and* not only depressive or anxiety but also psychotic symptoms [185,186]. Improving cognitive flexibility and attentional control may also help reduce “jumping-to-conclusion” tendencies (as in psychotic disorders) and emotional reactivity to stress [187,188], which may lessen both the activation and impact of negative affective states (e.g., low mood, anger, paranoia) and prior beliefs in the working memory [7]. Engagement in creativity, distraction tasks, and exercise are some techniques that may help improve attentional scope [189,190]. Recently, ecological momentary intervention, facilitated by smartphones, has offered promising directions in tackling patterns of ruminative thoughts in the everyday realm by supporting individuals in locating sources of distress and offering restorative tasks as distractions [191]. This may also be particularly useful as a prompt to reduce smartphone use and facilitate engagement in other behaviors to reduce rumination. The incorporation of the aforementioned elements in this novel intervention mode should be considered in future work. 

Given the differential capacity in emotional and cognitive processing across the age span, it could be expected that the factors that determine the degree of thought stagnation would considerably differ between young people and older adults. While several studies have found rumination to be less frequent among those of older age [192,193,194], further studies would be required to examine the specific mechanisms underlying rumination in this age group in greater depth, wherein different intervention modalities may be required. The present FMR, which takes external events, intentional activation, affect, and memory into account, may be adopted as a guide in such future studies.

## 6. Conclusions 

The current review re-examined ruminative constructs in relation to phenomenological experiences of associative thinking and streams of consciousness across psychiatric conditions. Moving away from compartmentalized approaches to understanding rumination as a form of self-focused cognition, the integrative Flow Model of Rumination bridges the influences of internal and external processes on rumination and accommodates a broad range of content and psychiatric conditions, thereby facilitating its application beyond depressive disorders as in the previous literature. 

Notably, the experience of rumination has understandably evolved in an unprecedented manner in the age of digitalization and global crises, which call for timely revisions to the conceptualization and understanding of the phenomenon. The ongoing global socio-political instabilities, as well as the rapid developments of digitalization and the emerging age of artificial intelligence, require individuals to continuously adjust to new modes of living, which has considerable impacts on cognition. Ultimately, rumination reflects a higher-level experience representing a disruption to the flow of thoughts. By recognizing the complexity underlying rumination and its related phenomena, a comprehensive framework such as the Flow Model of Rumination could help lay the foundation for future research and practice. Identifying individuals at risk of rumination not limited to depressive thought contents may also contribute to future developments of more holistic and efficacious targeted interventions.

## Data Availability

Not applicable.
